# Novel Predictive Nomogram for Identifying Difficult Guidewire Insertion in Patients With Malignant Colorectal Obstruction and Sphincterotome-Assisted Guidewire Insertion for Improving the Success Rate of Self-Expandable Metal Stent Insertion

**DOI:** 10.3389/fonc.2020.00637

**Published:** 2020-05-13

**Authors:** Zhenhua Zhu, Biming Li, Wangdi Liao, Nonghua Lv, Youxiang Chen, Xu Shu

**Affiliations:** Department of Gastroenterology, The First Affiliated Hospital of Nanchang University, Nanchang, China

**Keywords:** colorectal cancer, malignant colorectal obstruction, self-expandable metal stent, sphincterotome-assisted guidewire insertion, nomogram

## Abstract

**Aims:** This study aimed to identify risk factors related to guidewire insertion (GWI) failure and construct a novel predictive nomogram. In addition, sphincterotome-assisted guidewire insertion (SAGWI) in difficult cases was evaluated for efficacy and safety.

**Methods:** We reviewed the data of 509 patients with malignant colorectal obstruction who underwent endoscopic self-expandable metal stent (SEMS) insertion from 2007 to 2018 in our center, retrospectively. We identify risk factors associated with GWI failure by multivariate logistic regression analysis and construct a novel predictive nomogram. Improvements in the GWI and technical and clinical success rates were assessed for the SAGWI technique.

**Results:** A total of 509 patients with malignant colorectal obstruction were included. Increases of 6.9% and 7.0% were found in the GWI success rate by intention-to-treat (ITT; *p* < 0.001) and per-protocol (PP; *p* < 0.001) analyses after SAGWI, respectively. Increases of 6.5% and 6.6% in the technical success rate were found by ITT (*p* < 0.001) and PP (*p* < 0.001) analyses after SAGWI, respectively. Increases of 5.8% and 6.0% in the clinical success rate were found by ITT (*p* < 0.001) and PP (*p* < 0.001) analyses after SAGWI, respectively. Regarding the GWI failure-related factors, a sharply angulated stricture was an independent risk factor, and an experienced colonoscopist was an independent protective factor. A novel effective predictive nomogram was constructed.

**Conclusion:** The novel predictive nomogram can be conveniently used to identify difficult cases. A sharply angulated stricture and an experienced colonoscopist are independent factors related to GWI failure. The SAGWI technique is an effective and safe method for addressing technically difficult cases.

## Introduction

Malignant colonic obstruction occurs in about 7 to 29% of patients with complication of colorectal cancer (CRC) (1–4). This complication required surgical resection in the past. However, high morbidity and mortality rates will occur in emergency surgical decompression, with the highest reported rates reaching 60 and 22%, respectively (5). Additionally, temporary or permanent colostomy and ileostomy are often needed for surgical decompression, which impairs the quality of life.

Since the first colonic stent placement was reported, self-expandable metal stents (SEMSs) seem to be a good choice for emergency surgery in dealing with malignant colorectal obstruction (6). SEMSs can be used in palliative treatment or as a bridge to surgery to achieve single-stage surgery and reduce the need for colostomy. Most clinical studies have reported that SEMS placement is a safe single-stage preoperative treatment, while also improving clinical outcomes and quality of life for patients receiving palliative treatment (7–10). Consequently, SEMS has been widely accepted as a tool for initial treatment of obstructive CRC. Currently, SEMS placement with a combination of endoscope and fluoroscope techniques is preferred. The distal end of the lesion is located under the endoscope in this method, and the length and shape can be determined by injecting a water-soluble contrast. A stent is introduced by a guidewire, which goes through the working channel of the colonoscopy. Therefore, the introduction of the guidewire and SEMS placement can be completed under direct vision. However, a meta-analysis of seven randomized controlled trials (RCTs) on preoperative SEMS placement showed a mean technical success rate of only 76% (range, 47–100) (11). The difficulty of SEMS placement is due to the impossibility of passing through severe obstruction or sharply angulated strictures by guidewire (12–14). Thus, technical success is mainly dependent on the ability to pass a guidewire through the stricture. However, there are few data on the risk factors for guidewire insertion (GWI) failure. In addition, several case studies have reported that GWI in technically failed cases seems to be more successful with the use of a sphincterotome by rotating and bending its tip (15, 16). However, there is a lack of clinical studies on the efficacy and safety of this method.

In this study, we aimed to identify risk factors related to GWI failure and construct and validate a novel predictive nomogram for GWI failure. In addition, sphincterotome-assisted GWI (SAGWI) in difficult cases was evaluated for efficacy and safety.

## Patients and Methods

### Patients

An endoscopy database and clinical records from the First Affiliated Hospital of Nanchang University, Nanchang, China, were retrospectively reviewed. A total of 509 consecutive patients underwent SEMS placement for malignant obstruction between November 2007 and June 2018. Colorectal obstruction was confirmed by clinical symptoms and endoscopy or computed tomography (CT). Patients with bowel perforation or combined colorectal and small-bowel obstruction were excluded from the study. This study was approved by the Ethics Committee of the First Affiliated Hospital of Nanchang University.

### Endoscopic Technique

Before SEMS placement, all patients should undergo CT examination to assess the extent of the tumor; meanwhile, the site, degree, and length of the stricture were evaluated, as well as any other concurrent problems, such as perforation. The endoscopists of our hospital performed the SEMS placement. Enemas were performed in preparation for endoscopic bowel examination. All of the procedures were performed with a large working channel endoscope (CF-H260AI, Olympus, Tokyo, Japan, or EC-600WM, Fujifilm, Tokyo, Japan). A guidewire (Boston Scientific, Natick, MA) was used to pass through the stricture. Stents were selected by the endoscopist's preference and deployed with an extra 2-cm-longer stent on each side of the stricture.

In cases of failure to pass the guidewire through the stricture by using the conventional method (only guidewire), a sphincterotome (Boston Scientific, Natick, MA) with a preloaded guidewire was introduced through the working channel of the endoscope. The malignant stricture was cannulated by manipulating the sphincterotome with rotating and bending its tip under endoscopic vision. Then, the guidewire was introduced to pass through the stricture. The sphincterotome was removed after the appropriate position of the guidewire was placed. Then, the stent was introduced over the guidewire and deployed with covering the stricture (15).

The two most commonly used stent types in our hospital were as follows: (1) uncovered nitinol colonic stent (Micro-Tech, Nanjing, China); (2) uncovered WallFlex colonic stent (Boston Scientific, Denver, CO). The available WallFlex colonic stent lengths were 8 and 10 cm, with a 26-mm mid-body expansion diameter.

### Definitions

The definition of GWI failure was failure to pass the guidewire through the stricture. Cases with GWI failure by the conventional method (only guidewire) were included in the difficult GWI group (D-GWI group), and those with GWI success by the conventional method were included in the non-difficult GWI group (ND-GWI group). Cases in which SAGWI was applied were included in the SAGWI group.

The definition of technical failure was failure to deploy the stent through the stricture. The definition of clinical failure was absence of the disappearance of obstructive symptoms (abdominal pain, swollen abdomen, abdominal distension, vomiting, constipation, and so on) in spite of the achievement of technical success. Depending on the severity, obstruction was divided into total obstruction and subtotal obstruction. The definition of total obstruction was inability to pass any stool and gas, while subtotal obstruction was ability to pass only small amounts of liquid stool or gas. We ascertained whether carcinomatosis existed based on the CT scan. Carcinomatosis was defined as the implantation of tumor nodules along the peritoneal surface and contrast enhancement of the parietal peritoneal lining or loculated and/or septated ascitic fluid (17). The definition of experienced colonoscopist was a colonoscopist who had placed SEMSs in more than 100 cases.

### Outcomes and Statistical Analysis

The primary outcome of this study was to identify risk factors related to the failure of GWI by the conventional method and construct and validate a novel predictive nomogram for GWI failure. In addition, we compared the clinical outcomes of SEMS placement, including the rates of GWI, technical and clinical success, and complications between the D-GWI and ND-GWI groups. Furthermore, SAGWI in difficult cases was evaluated for efficacy and safety.

The results are expressed as the mean (± SD) or as a percentage. Continuous variables were analyzed by using Student's *t*-test and categorical variables using the χ^2^ test. Then, we performed multivariate logistic regression analysis to identify risk factors associated with the GWI failure by the conventional method. Then, a predictive model for GWI failure was constructed and presented as a nomogram to provide clinicians with an intuitive and quantitative tool for predicting GWI failure.

Statistical analysis was conducted with SPSS version 20.0 for Windows (SPSS, Inc., Chicago, IL) and R software version 3.1.3 (The R Foundation for Statistical Computing, www.R-project.org). The reported statistical significance levels were all two-sided, with significance set at 0.05.

## Results

### Patient Characteristics

A total of 509 patients (288 male patients; mean age, 62.3 ± 16.1 years) with malignant obstruction were enrolled in this study, with 456 (89.6%) in the ND-GWI group and 53 (10.4%) in the D-GWI group. Obstructions were caused by primary CRC in 468 (91.9%) patients and by an extracolonic malignancy (ECM) in 41 (8.1%) patients. Sharply angulated strictures were present in 137 (26.9%) patients. Additionally, 190 (37.3%) patients had peritoneal seeding, 426 (83.7%) experienced total obstruction, 96 (18.9%) underwent emergency endoscopic SEMS placement, and 313 (61.5%) underwent SEMS placement by an experienced colonoscopist. The baseline patient clinical and endoscopic characteristics are summarized in Table 1. Compared with the ND-GWI group, the D-GWI group showed significantly higher rates of total obstruction (94.3 vs. 82.5%; *p* = 0.027), carcinomatosis (54.7 vs. 35.3%; *p* = 0.006), and sharply angulated strictures (66.0 vs. 22.4%; *p* < 0.001) and a significantly lower rate of experienced colonoscopists (22.6 vs. 60.0%; *p* < 0.001). The obstruction site was also significantly different between the two groups (*p* = 0.038).

**Table 1:** Baseline characteristics of patients with malignant colorectal obstruction.

**Variable**	**Total**	**ND-GWI group**	**D-GWI group**	***p***
	***N* = 509**	***N* = 456**	***N* = 53**	
Sex, n (%)				0.556
Male	288 (56.6)	256 (56.1)	32 (60.4)	
Female	221 (43.4)	200 (43.9)	21 (39.6)	
Age, mean (SD), years	62.3 ± 16.1	62.2 ± 16.2	63.7 ± 15.3	0.512
Emergency endoscopic SEMS, *n* (%)				0.457
Yes	96 (18.9)	84 (18.4)	12 (22.6)	
No	413 (81.1)	372 (81.6)	41 (77.4)	
Anesthesia endoscopic SEMS, *n* (%)				0.976
Yes	57 (11.2)	51 (11.2)	6 (11.3)	
No	452 (88.8)	405 (88.8)	47 (88.7)	
Degree of obstruction, *n* (%)				0.027
Total	426 (83.7)	376 (82.5)	50 (94.3)	
Subtotal	83 (16.3)	80 (17.5)	3 (5.7)	
Obstruction site, *n* (%)				0.038
Rectum	124 (24.4)	118 (25.9)	6 (11.3)	
Sigmoid colon	202 (39.7)	168 (36.8)	28 (52.8)	
Descending colon	85 (16.7)	78 (17.1)	7 (13.2)	
Transverse colon	67 (13.2)	57 (12.5)	10 (18.9)	
Ascending colon	37 (7.3)	35 (7.7)	2 (3.8)	
Sharply angulated stricture, *n* (%)				<0.001
Presence	137(26.9)	102(22.4)	35(66.0)	
Absence	372 (73.1)	354 (77.6)	18 (34.0)	
Etiology, *n* (%)				0.356
Colorectal malignancy	468 (91.9)	421 (92.3)	47 (88.7)	
Extracolonic malignancy	41 (8.1)	35 (7.7)	6 (11.3)	
Carcinomatosis, *n* (%)				0.006
Presence	190 (37.3)	161 (35.3)	29 (54.7)	
Absence	319 (62.7)	295 (64.7)	24 (45.3)	
Organ metastasis				0.236
Presence	109 (21.4)	101 (22.1)	8 (15.1)	
Absence	400 (78.6)	355 (77.9)	45 (84.9)	
Experienced colonoscopist, *n* (%)				<0.001
Yes	313 (61.5)	301 (66.0)	12 (22.6)	
No	196 (38.5)	155 (34.0)	41 (77.4)	

*ND-GWI group, non-difficult guidewire insertion group; D-GWI, difficult guidewire insertion group*.

*Experienced colonoscopists had placed self-expandable metal stents in more than 100 cases*.

### Clinical Outcomes

**Figure 1:**
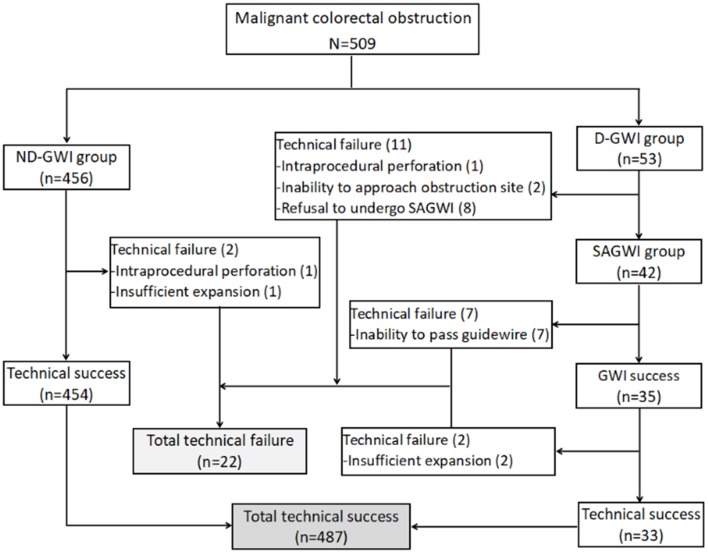
Study flowchart. GWI, guidewire insertion; ND-GWI group, non-difficult guidewire insertion group; D-GWI, difficult guidewire insertion group; SAGWI group, sphincterotome-assisted guidewire insertion group.

Figure 1 and Table 2 show the study flowchart and clinical outcomes of the SEMS. In the D-GWI group, 42 patients (42/53; 79.2%) underwent SAGWI, while 11 patients did not for the following reasons: intraprocedural perforation (one patient), inability to approach the obstruction site (two patients), and refusal to undergo SAGWI (eight patients). By the intention-to-treat (ITT) and per-protocol (PP) analyses, the total GWI success rates were 96.5% (491/509) and 98.6% (491/498), respectively; the technical success rates were 95.7% (487/509) and 97.8% (487/498), respectively; and the clinical success rates were 91.9% (468/509) and 94.0% (468/498), respectively. Twenty-two patients with technical failure underwent emergency surgery. The rates of GWI, technical, and clinical success were significantly different between the two study groups except for complications. Among 509 patients who underwent SEMS placement, a total of 24 (4.7%) patients experienced complications. Bleeding (*n* = 14 patients) was the most common complication, followed by reobstruction (*n* = 7), perforation (*n* = 2), and stent migration (*n* = 1). The complication rate was slightly higher in the D-GWI group but did not show a significant difference (7.5 vs. 4.4%; *p* = 0.304).

**Table 2:** Clinical outcomes of self-expandable metal stent insertion.

**Variable**	**Total**	**ND-GWI group**	**D-GWI group**	***p***
	***N* = 509**	***N* = 456**	***N* = 53**	
Guidewire insertion success, *n*/*N* (%)				
ITT analysis	491/509 (96.5)	456/456 (100)	35/53 (66.0)	<0.001
PP analysis	491/498 (98.6)	456/456 (100)	35/42 (83.3)	<0.001
Technical success, *n* (%)				
ITT analysis	487/509 (95.7)	454/456 (99.7)	33/53 (62.3)	<0.001
PP analysis	487/498 (97.8)	454/456 (99.7)	33/42 (78.6)	<0.001
Clinical success, *n* (%)				
ITT analysis	468/509 (91.9)	438/456 (96.1)	30/53 (56.6)	<0.001
PP analysis	468/498 (94.0)	438/456 (96.1)	30/42 (71.4)	<0.001
Complication, n (%)	24/509 (4.7)	20/456 (4.4)	4/53 (7.5)	0.304
Perforation	2/509 (0.4)	1/456 (0.2)	1/53 (1.9)	–
Reobstruction	7/509 (1.4)	6/456 (1.3)	1/53 (1.9)	–
Stent migration	1/509 (0.2)	1/456 (0.2)	0	–
Bleeding	14/509 (2.7)	12/456 (2.6)	2/53 (3.8)	–

*ND-GWI group, non-difficult guidewire insertion group; D-GWI, difficult guidewire insertion group; ITT, intention-to-treat analysis; PP, per-protocol analysis*.

**Figure 2:**
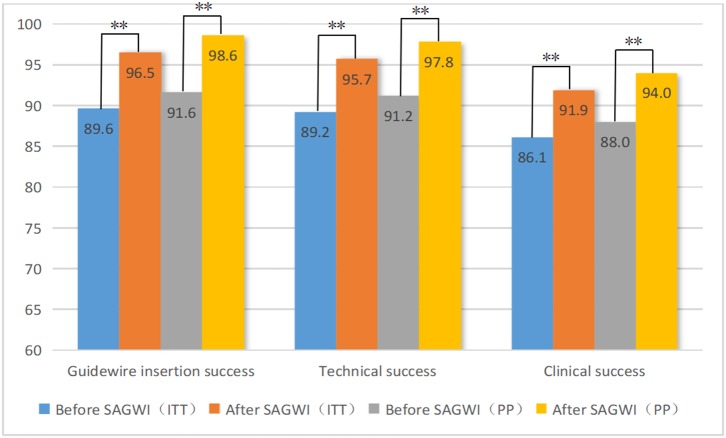
Rates of guidewire insertion, technical, and clinical success before and after SAGWI in 509 patients with malignant colorectal obstruction. SAGWI, sphincterotome-assisted guidewire insertion; ITT, intention-to-treat analysis; PP, per-protocol analysis. ** *p* < 0.001.

Figure 2 shows the rates of GWI, technical, and clinical success in the 509 patients before and after SAGWI. Increases of 6.9% and 7.0% were found in the GWI success rate by ITT (89.6 vs. 96.5%; *p* < 0.001) and PP (91.6 vs. 98.6%; *p* < 0.001) analyses after SAGWI, respectively. Increases of 6.5% and 6.6% in the technical success rate were found by ITT (89.2 vs. 95.7%; *p* < 0.001) and PP (91.2 vs. 97.8%; *p* < 0.001) analyses after SAGWI, respectively. Increases of 5.8 and 6.0% in clinical success rate were found by ITT (86.1 vs. 91.9%; *p* < 0.001) and PP (88.0 vs. 94.0%; *p* < 0.001) analyses after SAGWI, respectively.

### Risk Factors and Predictive Nomogram for GWI Failure

**Figure 3:**
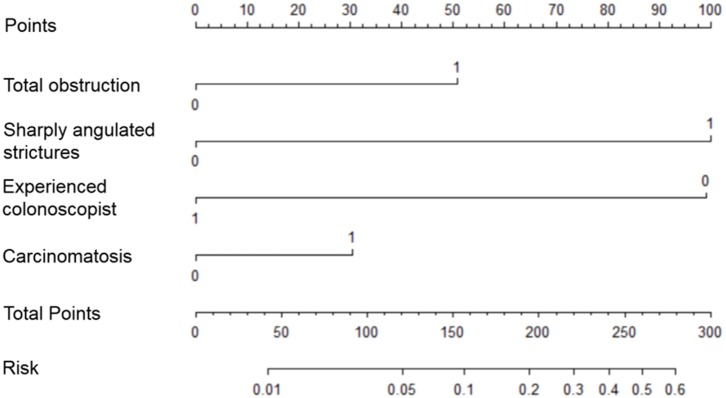
Predictive nomogram for guidewire insertion failure.

**Figure 4:**
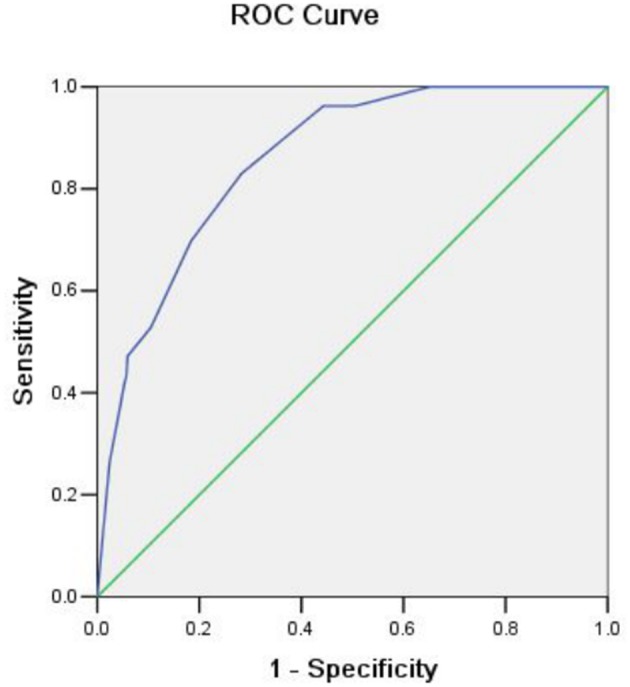
Receiver operating characteristic (ROC) curve showing the predictive ability for guidewire insertion failure.

Risk factors influencing GWI failure were analyzed by multivariate logistic regression analysis (Table 3). A sharply angulated stricture [odds ratio (OR) = 8.345, 95% confidence interval (CI): 4.226–16.480, *p* < 0.001] was identified as an independent risk factor for GWI failure, and an experienced colonoscopist (OR = 0.130, 95% CI: 0.063–0.271, *p* < 0.001) was identified as an independent protective factor. A predictive model of GWI failure was constructed based on the multivariate logistic regression analysis results, as follows: logit*P* = −3.413 + 1.027 × total obstruction + 2.122 × sharply angulated stricture + 0.628 × carcinomatosis – 2.037 × experienced colonoscopist. A predictive nomogram based on the predictive model was developed (Figure 3). The predictive ability of the model and nomogram was analyzed by receiver operating characteristic (ROC) curve analysis. The area under the ROC curve (AUC) was 0.858 (95% CI: 0.814–0.902). The specificity, sensitivity, and accuracy of the predictive model were 0.717, 0.830, and 0.774, respectively, when the cutoff value was 0.877 (Table 4, Figure 4).

**Table 3:** Independent predictors for the failure of guidewire insertion by multivariate analysis.

**Variable**	***B***	**SE**	**Wald**	**OR (95% CI)**	***p*-value**
Total obstruction	1.071	0.661	2.623	2.918 (0.799–10.661)	0.105
Sharply angulated stricture	2.107	0.342	37.935	8.220 (4.205–16.071)	<0.001
Carcinomatosis	0.643	0.332	3.744	1.902 (0.992–3.648)	0.053
Experienced colonoscopist	−2.090	0.371	31.688	0.124 (0.060–0.256)	<0.001
Constant	−3.397	0.688	24.404	0.033	<0.001

*OR, odds ratio; CI, confidence interval*.

**Table 4:** Predictive ability of the model.

	**ROC: AUC (95% CI)**	***p*-value**	**Best threshold**	**Specificity**	**Sensitivity**	**Accuracy**
Predictive model	0.858 (0.814–0.902)	<0.001	0.877	0.717	0.830	0.774

*ROC, receiver operating characteristic; AUC, area under the ROC curve*.

## Discussion

**Figure 5:**
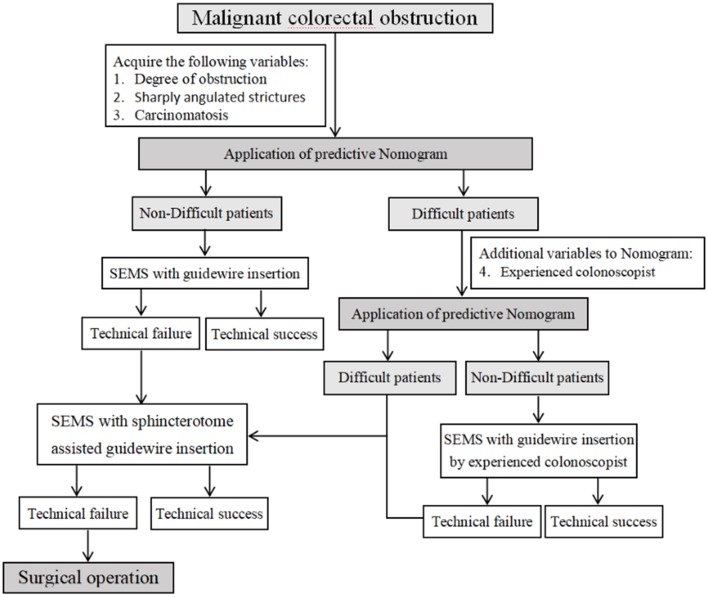
Algorithm for treating malignant colorectal obstruction with self-expandable metal stent placement.

For all we know, this is the first study to focus on GWI failure and construct a predictive nomogram. In addition, the SAGWI technique was evaluated for efficacy and safety in difficult cases and may be a useful and effective technique for addressing difficult cases, especially for inexperienced colonoscopists. We analyzed the factors associated with GWI failure based on a large-scale, detailed dataset from patients with malignant colonic obstruction in our hospital. After multivariate logistic regression analysis, a sharply angulated stricture was determined as an independent risk factor of GWI failure, and an experienced colonoscopist was an independent protective factor. In addition, we constructed an effective predictive model and nomogram for GWI failure with four variables: total obstruction, sharply angulated stricture, carcinomatosis, and experienced colonoscopist. Based on the above results, we proposed an algorithm for treating malignant colorectal obstruction with SEMS placement (Figure 5). When we encounter patients with malignant colorectal obstruction, we first perform assessments for total obstruction, sharply angulated strictures, and carcinomatosis to be evaluated by the nomogram. If the case is considered difficult, we add an additional indicator (experienced colonoscopist) to be reevaluated, and if the case is still considered difficult, the SAGWI technique could be introduced. If the case is not considered difficult, routine GWI is considered first by an experienced or inexperienced colonoscopist based on the nomogram evaluation. After routine GWI failure, the SAGWI technique is considered.

A recent multicenter retrospective study from Korea reported 90.5% technical success rates and 81.0% clinical success rates (18), and another single-center study of patients with malignant large-bowel obstruction from an extracolonic malignancy reported lower technical and clinical success rates of 75.9 and 54.5%, respectively (19). A previous meta-analysis of seven RCTs on preoperative SEMS placement reported a mean technical success rate of only 76% (range, 47–100) (11). In our study, the technical and clinical success rates were 89.2% (454/509) and 86.1% (438/509) by ITT analysis and 91.2% (456/498) and 88.0% (438/498) by PP analysis, respectively, before the use of the SAGWI technique, which is similar to those of previous studies. When the SAGWI technique was introduced to address technical failure, 6.9 and 7.0% increases were obtained in the GWI success rate by ITT (89.6 vs. 96.5%; *p* < 0.001) and PP (91.6 vs. 98.6%; *p* < 0.001) analyses after SAGWI, respectively. Therefore, the technical and clinical success rates increased to 95.7% (487/509) and 91.9% (468/509) by ITT analysis and 97.8% (487/498) and 94.0% (468/498) by PP analysis, respectively. A 5.8–6.6% improvement in the technical or clinical success rate was achieved (*p* < 0.001).

When the lumen past the lesion is angulated relative to the endoscopic view, the position of the small lumen on a bend can render cannulation of the stricture difficult. In our study, sharply angulated strictures were present in 137 patients (26.9%), and the rate of sharply angulated strictures (66.0 vs. 22.4%; *p* < 0.001) was significantly higher in the D-GWI group. A sphincterotome with torque control allowed precise angular and rotational adjustment of the guidewire as it engaged the stricture. Therefore, the D-GWI group showed 66.0% (35/53) and 83.3% (35/42) technical success rates by ITT and PP analyses, respectively. The rates of total obstruction (94.3 vs. 82.5%; *p* = 0.027) and carcinomatosis (54.7 vs. 35.3%; *p* = 0.006) were significantly higher in difficult cases. Bowel immobilization caused by peritoneal carcinomatosis may have resulted in the increased technical failure of SEMS placement. Anesthesia and emergency endoscopy did not affect the GWI success rate. Obstructions caused by an extracolonic malignancy were less frequent than that in a previous study (8.1 vs. 24.5%) (18), but the rate of total obstruction was slightly higher (83.7 vs. 75.5%). A total of 313 SEMSs were placed by an experienced colonoscopist in our study, with GWI success in 96.2% (303/313) of cases; this rate is higher than that for SEMSs placed by an inexperienced colonoscopist [78.1% (155/196), *p* < 0.001]. An experienced colonoscopist was identified as a protective factor for GWI by multivariate logistic regression analysis, which indicates that the experience of the colonoscopist has a great influence on technical failures. We think that more experienced colonoscopists are more familiar with the colonoscopic approach and manipulation of the guidewire. The obstruction site was more frequent in the sigmoid colon in difficult cases (*p* = 0.038), similar to a previous study (18). The overall complication rate of this study was 4.7%, which is slightly lower than that of a previous study (7.2%) (20). This may be due in part to the fact that 61.5% (313/509) of the patients were treated by an experienced colonoscopist. The complication rate of the two groups was similar (ND-GWI group vs. D-GWI group: 4.4 vs. 7.5%, *p* = 0.304), and bleeding was the most frequent complication; these results indicate that SAGWI is a safe technique.

The present study has some limitations. First, this a retrospective study, which could introduce bias. Second, this is a clinical study performed in a single tertiary-care center and with different colonoscopists with various levels of experience. Finally, follow-up data were insufficient to evaluate long-term complications.

In conclusion, the present study had a large sample size and representative results and performed a comprehensive analysis. As far as we know, this is the largest study to specifically identify factors associated with GWI failure in patients with malignant colonic obstruction in whom SEMS placement was performed. In addition, we constructed a predictive model based on the multivariate logistic regression analysis results, and this model was displayed as a nomogram to provide clinicians with an intuitive and quantitative tool for predicting GWI failure, which may be practical for clinical use. Furthermore, the SAGWI technique is an effective and safe method for addressing technically difficult cases and improving the technical success rate.

## Data Availability Statement

The datasets generated for this study are available on request to the corresponding author.

## Ethics Statement

This study was approved by the institutional review board of the First Affiliated Hospital of Nanchang University.

## Author Contributions

ZZ and BL acquisition of data, analysis and interpretation of data, statistical analysis, and drafting of the manuscript. WL and NL acquisition of data, administrative, technical, or material support. YC and XS study concept and design, revision of the manuscript for important intellectual content, study supervision. All authors approved the final version of the manuscript.

## Conflict of Interest

The authors declare that the research was conducted in the absence of any commercial or financial relationships that could be construed as a potential conflict of interest.
